# Supplementary material for: Identifying SARS-CoV-2 Variants of Concern through saliva-based RT-qPCR by targeting recurrent mutation sites
Source: medRxiv. 2022 Mar 4:2022.03.02.22271785. Preprint. [Version 1] doi: 10.1101/2022.03.02.22271785 (PMC8902870; doi:10.1101/2022.03.02.22271785)
Supplement: 7 [file NIHPP2022.03.02.22271785v1-supplement-7.pdf]

medRxiv preprint doi: https://doi.org/10.1101/2022.03.02.22271785; this version posted March 4, 2022. The copyright holder for this preprint
(which was not certified by peer review) is the author/funder, who has granted medRxiv a license to display the preprint in perpetuity.
It is made available under a CC-BY 4.0 International license .

Supplemental Table 1. Reaction efficiencies for all primer and probe sets in multiplex.

RT-qPCR Assay	Multiplex Reaction Efficiency		
	Internal Control Probe	Reference Probe	Deletion/Substitution Probe
SΔ69-70	95.49%	100.57%	97.12%
ORF1a Δ3675-3677	101.56%	95.63%	101.44%
K417T	N/A	101.97%	89.52%
E484K	N/A	99.04%	102.19%
E484Q	N/A	94.69%	101.40%
L452R	N/A	103.99%	112.04%

Supplemental Table 2. Thermocycling Conditions for RT-qPCR assays.

SΔ69-70 and ORF1aΔ3675-3677			
**Stage**	**Temperature (°C)**	**Duration**	**Cycles**
Warm Up	25.0	2 min	1
Reverse Transcription	50.0	15 min	1
Initial Denaturation	95.0	2 min	1
Touchdown	95.0	3 sec	3
	72.0	30 sec	
	95.0	3 sec	3
	68.0	30 sec	
	95.0	3 sec	3
	64.0	30 sec	
Main Amplification	95.0	3 sec	45
	60.0	30 sec	
TaqPath SNP Assays (K417T, E484K, E484Q, and L452R)			
**Stage**	**Temperature (°C)**	**Duration**	**Cycles**
Warm Up	25.0	2 min	1
Reverse Transcription	50.0	15 min	1
Initial Denaturation	95.0	2 min	1
Main Amplification	95.0	3 sec	45
	60.0	30 sec	

medRxiv preprint doi: https://doi.org/10.1101/2022.03.02.22271785; this version posted March 4, 2022. The copyright holder for this preprint (which was not certified by peer review) is the author/funder, who has granted medRxiv a license to display the preprint in perpetuity.
It is made available under a CC-BY 4.0 International license .

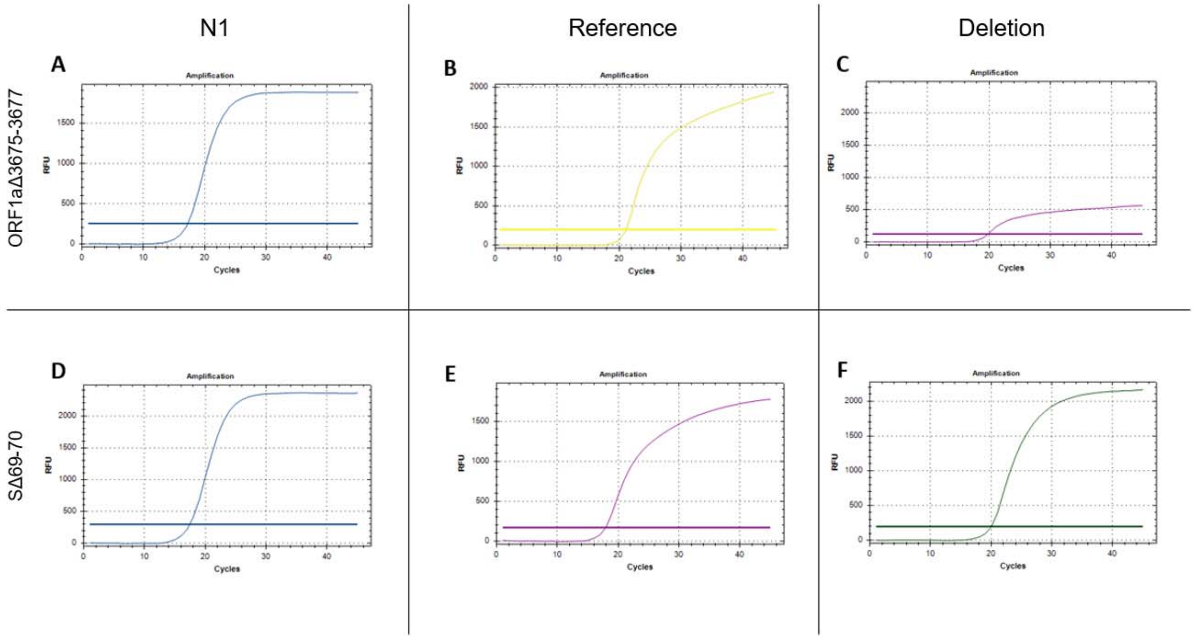

Supplemental Figure 1. Representative curves of RT-qPCR deletion assays.

medRxiv preprint doi: https://doi.org/10.1101/2022.03.02.22271785; this version posted March 4, 2022. The copyright holder for this preprint (which was not certified by peer review) is the author/funder, who has granted medRxiv a license to display the preprint in perpetuity.
It is made available under a CC-BY 4.0 International license .

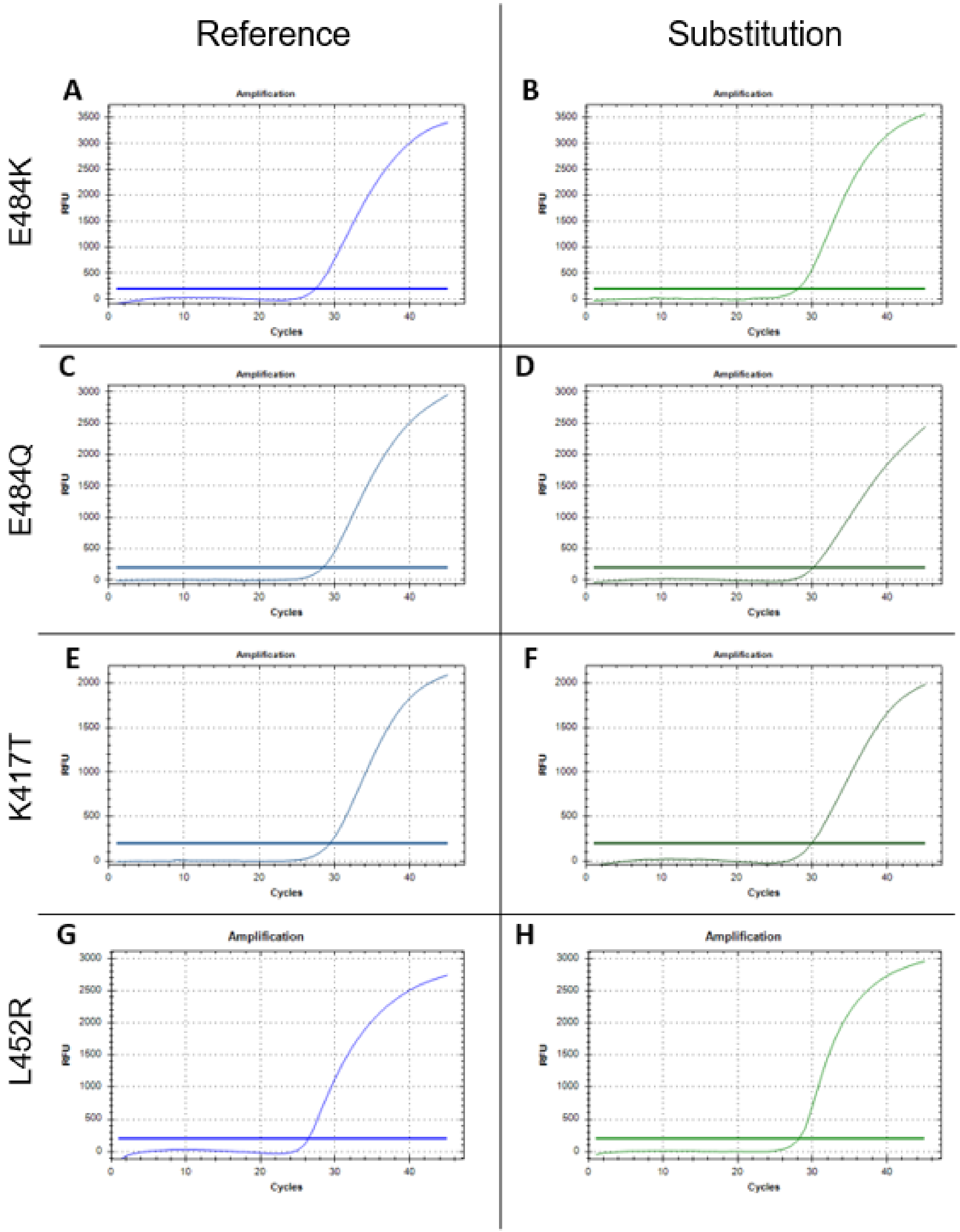

Supplemental Figure 2. Representative curves of TaqPath Spike SNP assays.

medRxiv preprint doi: https://doi.org/10.1101/2022.03.02.22271785; this version posted March 4, 2022. The copyright holder for this preprint (which was not certified by peer review) is the author/funder, who has granted medRxiv a license to display the preprint in perpetuity.
It is made available under a CC-BY 4.0 International license .

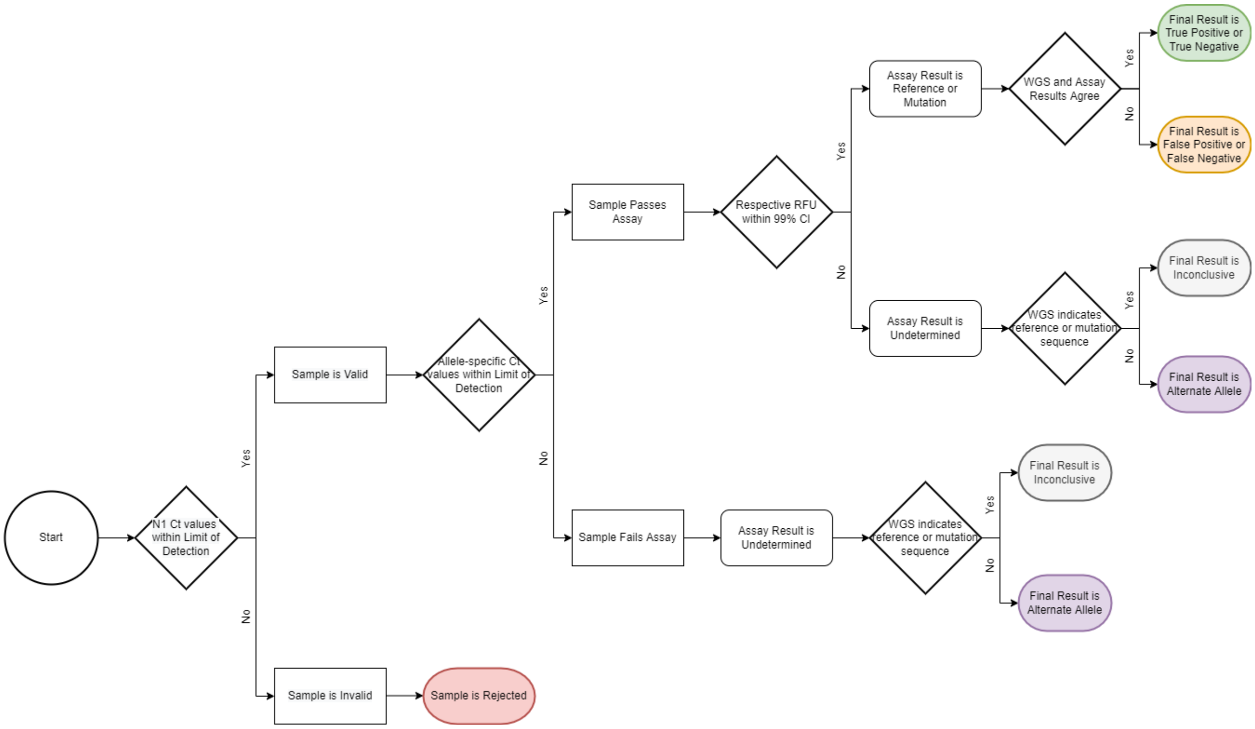

Supplemental Figure 3. Sample Resulting Flowchart.
